# Supplementary material for: Elevated ETV6 Expression in Glioma Promotes an Aggressive In Vitro Phenotype Associated with Shorter Patient Survival
Source: Genes (Basel). 2022 Oct 17;13(10):1882. doi: 10.3390/genes13101882 (PMC9656946; doi:10.3390/genes13101882)
Supplement: Supplementary file 1 [file genes-13-01882-s001.zip › genes-1945896-supplementary.pdf]

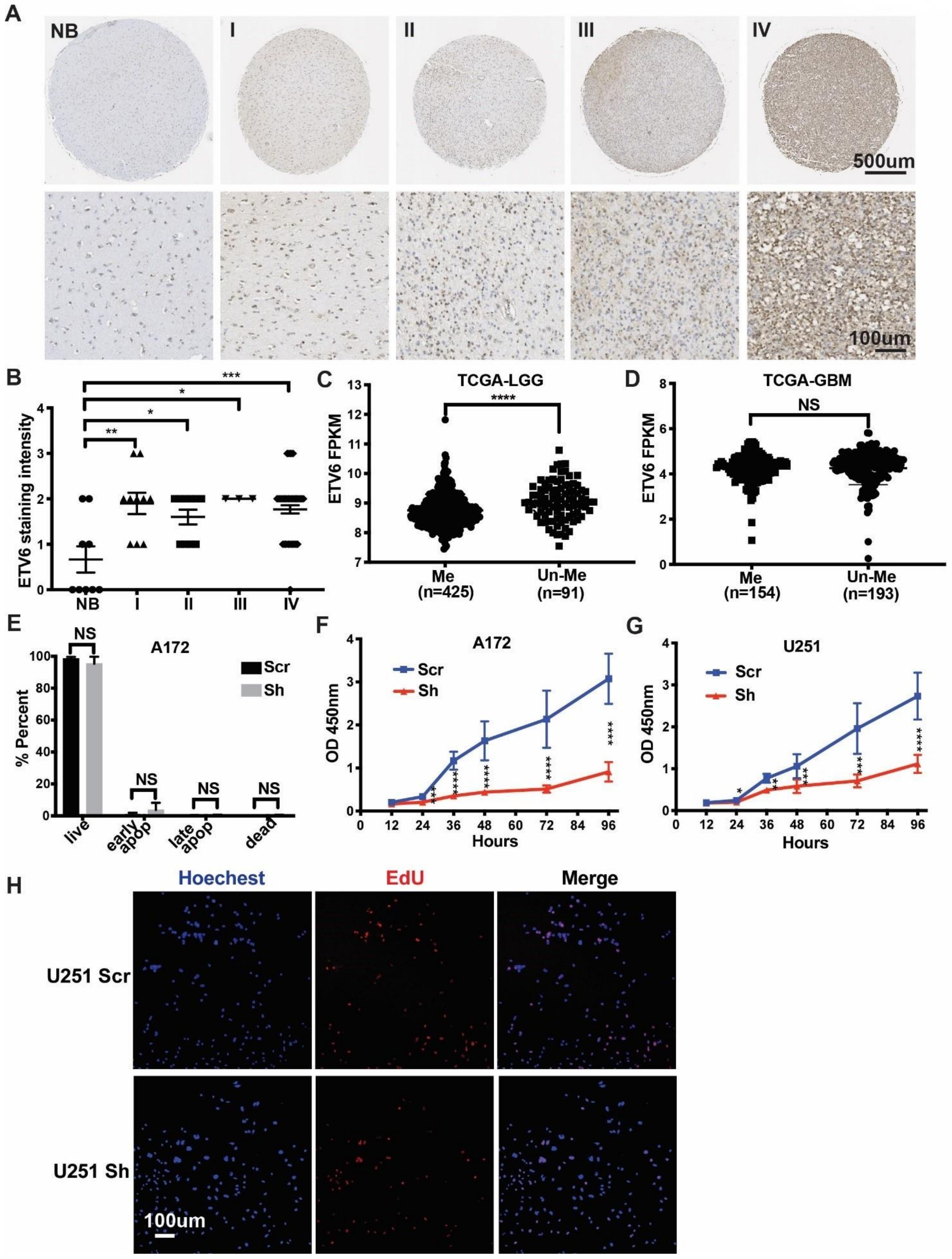

Figure S1. ETV6 expression in glioma and its effect on glioma apoptosis and proliferation. (A) Representative images of ETV6 staining in normal brain, grade I-IV glioma from tissue microarrays (US Biomax, cat#BS17017b, and GL805bt) are shown. Scale bar, 500μm for upper row; 100μm for lower row. (B) The staining intensity of ETV6 was scored as 0-3 on normal brain and grade I-IV glioma (n=9, 10, 10, 4, 65, respectively). ETV6 expression in grade I-IV glioma was significantly

higher than those in normal brains. (C-D) The association of ETV6 mRNA expression and MGMT promoter methylation (Me) or unmethylation (Un-Me) status was assessed in LGG and GBM from TCGA, respectively. n=425 vs. n=91 for Me vs. Un-Me in LGG; n=154 vs. n=193 for Me vs. Un-Me in GBM. (E) ETV6 knockdown by shRNA had little effect on apoptosis in A172 cells as revealed by AnnexinV-PE and 7-AAD flow cytometric analysis. (F-G) CCK8 assay showed cell growth was attenuated significantly as early as 24hrs after plating in shETV6 infected A172 and U251 cells, respectively. (H) EdU assay revealed that ETV6 knockdown by shRNA had little effect on proliferation in U251 cells. Scale bar, 100μm (*, $p < 0.05$; **, $p <0.01$; ***, $p < 0.001$; ****, $p < 0.0001$).
